# Brazilian Protocol for Sexually Transmitted Infections, 2020: infections that cause urethral discharge

**DOI:** 10.1590/0037-8682-633-2020

**Published:** 2021-05-17

**Authors:** Leonor Henriette de Lannoy, Roberto José de Carvalho da Silva, Edilbert Pellegrini Nahn, Eduardo Campos de Oliveira, Pâmela Cristina Gaspar

**Affiliations:** 1 Ministério da Saúde, Secretaria de Vigilância em Saúde, Brasília, DF, Brasil.; 2 Universidade Federal do Espírito Santo, Programa de Pós-Graduação em Infectologia, Vitória, ES, Brasil.; 3 Faculdade das Américas, Centro Universitário, São Paulo, SP, Brasil.; 4 Secretaria de Estado da Saúde de São Paulo, Programa Estadual de DST/Aids, São Paulo, SP, Brasil.; 5 Universidade Federal do Estado do Rio de Janeiro, Campus Macaé, RJ, Brasil.; 6 Faculdade de Medicina de Campos, Campos dos Goytacazes, RJ, Brasil.; 7 Secretaria de Estado da Saúde de Santa Catarina, Gestão Estadual de DST/Aids/HIV de Santa Catarina, Florianópolis, SC, Brasil.; 8 Universidade de Brasília, Programa de Pós-Graduação em Saúde Coletiva, Brasília, DF, Brasil.

**Keywords:** Urethritis, Neisseria gonorrhoeae, Chlamydia trachomatis, Clinical protocols, Public health

## Abstract

This article approaches infections that cause urethral discharge. This theme is part of the Clinical Protocol and Therapeutic Guidelines for Comprehensive Care for People with Sexually Transmitted Infections, published by the Ministry of Health of Brazil in 2020. These guidelines were prepared based on scientific evidence and validated in discussions with experts. Urethritis can cause severe and even irreversible health damage when not properly treated, or when the microorganism develops antimicrobial resistance. It is noteworthy that the high levels of antimicrobial resistance grown by pathogens that cause urethritis comprises a global emergency in public health. This article presents epidemiological and clinical aspects, recommendations on diagnostic and treatment, and strategies for surveillance, prevention, and control actions for infections that cause urethral discharge, to contribute to managers' and health professionals' care qualification.

## Highlighted excerpt

Associated factors with urethritis are: young age, low socioeconomic level, multiple partnerships or new sexual partnership, history of STI, irregular use of condoms, and lack of access to adequate diagnosis and treatment.

## INTRODUCTION

The article addresses infections that cause urethral discharge. This subject composes the Clinical Protocol and Therapeutical Guidelines (PCDT) for Comprehensive Care for People with Sexually Transmitted Infections (STIs), published by the Health Surveillance Secretariat of the Brazilian Ministry of Health. For elaborating the PCDT, evidences available in the literature were selected, analysed and discussed by a panel of experts. The referred PCDT was approved by the National Committee for the Incorporation of Technologies in the Brazilian National Health System (Conitec)^1^ and updated by the team of experts in STI in 2020.

## EPIDEMIOLOGICAL ASPECTS

Urethritis is defined as an inflammation of the urethra and can be infectious or not. Several agents can cause infectious urethritis in the context of condom-free sexual practices. Urethritis is classified according to the etiologic agent as gonococcal, caused by *Neisseria gonorrhoeae*, and non-gonococcal, caused mainly by *Chlamydia trachomatis* and *Mycoplasma genitalium*. Other agents, such as *Trichomonas vaginalis*, enterobacteria (in insertive anal relations), herpes simplex virus (HSV), adenovirus, and *Candida sp.*, are less frequent^2^ ^,^ ^3^.

The following factors are associated with urethritis: young age, low socioeconomic status, multiple partnerships or new sexual partnerships, history of STI and irregular condom use, and lack of access to adequate diagnosis and treatment^2^ ^,^ ^3^.

The World Health Organization (WHO), through a systematic review study, estimated for the year 2016 the occurrence of 370.4 million new curable urogenital infections by chlamydia, gonorrhea, and trichomoniasis in women and men aged 15 to 49^4^. The global incidence rate of chlamydia in 2016 was 34 cases per 1,000 women and 33 cases per 1,000 men; of gonorrhea, 20 cases per 1,000 women and 26 cases per 1,000 men; and of trichomoniasis, 40 cases per 1,000 women and 42 cases per 1,000 men^4^. A systematic review and meta-analysis study on the prevalence of *M. genitalium*, which included three studies with 3,809 people, estimated this agent’s prevalence at 1.3% in developed countries and 3.9% in developing countries. The prevalence was similar in men and women^5^. Analyses of the European Centre for Disease Prevention and Control (ECDC) found that in 2018, among 17 countries that collected data on the mode of transmission of gonorrhea, 48% of all confirmed and reported cases occurred in men who have sex with men^6^.

In Brazil, the epidemiological scenario of infections that cause urethral discharge follows the high worldwide rates. It is estimated that the prevalence of gonorrhea in the population between 15 and 49 years of age is approximately 1.4%. The incidence in the general population is around 500,000 new cases per year^7^.

Regarding the risk of transmission, *N. gonorrhea* has a chance of transmission from infected man to woman varying from 50% to 73%, regardless of the number of exposures. A man's chance of becoming infected from an infected woman varies from 20% to 35% in an exposure^8^. Unprotected oral sex results in infection about 25% of the time since the pharynx is one of the pathogen's largest asymptomatic reservoirs^9^.

In the case of *C. trachomatis*, the probability of a man contracting the infection from a woman is 32%, and a woman contracting the infection from a man, 40%; this transmission proportion refers to contact with an infected partner through unprotected sex^10^. Although it is well established that *M. genitalium* is sexually transmitted, it is not known how many times this occurs per episode of unprotected intercourse^11^. Studies suggest that the transmission of *M. genitalium* is probably lower than that of *C. trachomatis*, consistent with these agents' infective load. Men with symptomatic non-gonococcal urethritis and presumably higher loads of *M. genitalium* are likely to be more infectious than men with asymptomatic infections^12^ ^,^ ^13^.

The risk of men becoming infected from partners infected with *T. vaginalis* ranges from 22% to 72%. Since there is contamination in up to 5% of people who report recent receptive anal sex, it is necessary to clarify whether the rectum may be a reservoir for *T. vaginalis* infection^14^. According to the Centers for Disease Control and Prevention (CDC), rectal and oral testing for *T. vaginalis* is not recommended due to the lack of evidence of such infections at these sites.

**FIGURE 1:**
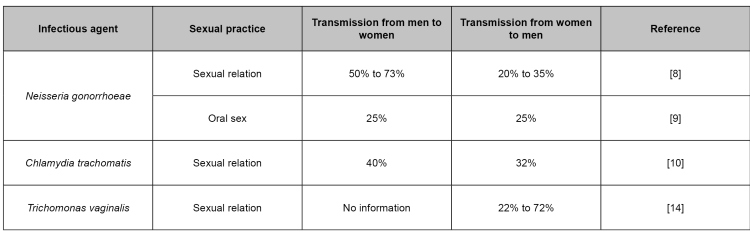
Risk of transmission of infectious agents that cause urethral discharge in unprotected sexual practices.

Figure 1 presents a summary of risks offered by each infectious agent that cause urethral discharge.

## CLINICAL ASPECTS

Urethral discharge is a clinical syndrome characterized by the appearance of discharge ranging from mucoid to purulent, with variable volume, which may be associated with urethral pain (regardless of urination), dysuria, stranguria (slow and painful urination), urethral pruritus, and urethral meatus erythema. Purulent urethral discharge corresponds to 75% of urethritis caused by *N. gonorrhoeae* and 11% to 33% of non-gonococcal urethritis. In contrast, the mucoid aspect is seen in about 25% of gonococcal urethritis cases and 50% of non-gonococcal urethritis cases^2^.

Gonococcal urethritis is an infectious and inflammatory process of the urethral mucosa. After the incubation period, which lasts an average of two to five days, ranging from one to 10 days, the infection progresses symptomatically. Dysuria is defined as the sensation of tingling and intraurethral itching followed by pain at urination. The discharge, initially mucoid, becomes purulent in one to two days, with larger volume and accompanied by urethral meatus edema. Some men may present fever, as well as manifestations of an acute systemic infection. About 95% of cases become asymptomatic in three months, and a proportion of untreated patients may evolve to spontaneous healing in a few weeks^15^.

The clinical complications from not receiving immediate treatment occur in up to 50% of the cases due to infection progression to the posterior urethra. Acute epididymitis is the most frequent complication and may evolve with epididymal duct's obstruction, determining oligozoospermia, azoospermia, and sterility. Other consequences of gonococcal urethritis are prostatitis, orchitis, penile edema (particularly of the foreskin), balanoposthitis, and also lesions of the sebaceous and acinar glands of the genital region, tysonitis (Tyson glands), cowperitis (Cowper glands), and littritis (Littré glands)^15^.

Non-gonococcal urethritis is symptomatic urethritis whose bacterioscopy by Gram staining, culture, and molecular detection of specific nucleic acid is negative for gonococcus. Human chlamydia infection is responsible for approximately 50% of cases of non-gonococcal urethritis. The incubation period in humans is 14 to 21 days. It is estimated that two-thirds of stable female partners of men with non-gonococcal urethritis host *C. trachomatis* in the endocervix, thus being able to reinfect their sexual partners and evolve into pelvic inflammatory disease if no treatment is carried out^16^ ^-^ ^18^. 

In men, non-gonococcal urethritis is usually characterized by discrete mucoid discharge, with slight and intermittent dysuria. Subacute urethritis is the form of presentation in approximately 50% of men with urethritis caused by *C. trachomatis* ^19^. However, in some cases, discharges from non-gonococcal urethritis can clinically simulate those from gonorrhea. Urethritis caused by *C. trachomatis* may evolve into prostatitis, epididymitis, balanitis, conjunctivitis (by autoinoculation), urethral-conjunctive-synovial syndrome, or Reiter's syndrome.

Women with urethritis present mild dysuria, which may be accompanied by vaginal discharge or bleeding. Physical examination may reveal the presence of mucopurulent cervicitis or even vulvovaginal herpetic-like lesions^20^.

In persistent urethritis cases, the evaluation must be performed mainly through clinical history, considering the possibility of reinfection or inadequate treatment for chlamydia and gonorrhea. If such situations are ruled out, agents not susceptible to the previous treatment (e.g., *M. genitalium* and *T. vaginalis*), as well as the occurrence of antimicrobial resistance, must be investigated^2^. 

Other non-infectious causes of urethritis, such as trauma (constant urethral milking), instrumentation, insertion of intraurethral or paraurethral foreign bodies (piercings), and chemical irritation (use of lubricants and spermicides), must be considered in the differential diagnosis of persistent urethritis^2^. 

## DIAGNOSIS

**FIGURE 2:**
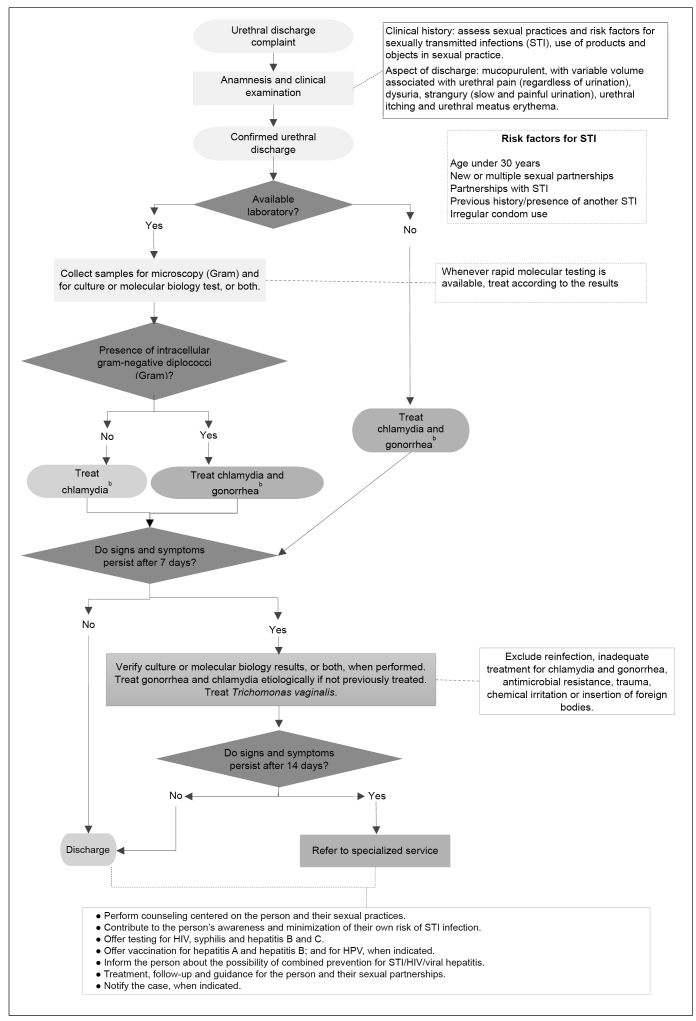
Recommendations for management of urethral discharge.

**Source:** adapted from the Clinical Protocol and Therapeutic Guidelines for Comprehensive Care to People with Sexually Transmitted Diseases, 2020^2^.

**Notes:** a) There are molecular biology kits that detect more pathogens simultaneously, besides chlamydia and gonococcus, and that are also useful for the etiological diagnosis of urethritis, such as *M. genitalium*; b) The therapeutic protocol also covers treatment for *M. genitalium.*

The treatment of urethral discharge can be performed through syndromic management for situations where there is no laboratory support^2^ ^,^ ^21^ ^,^ ^22^(Figure 2). For cases of male urethral discharge, it is estimated that the syndromic management has a sensitivity ranging from 84% to 95%^23^.

The use of diagnostic tests is indicated for screening asymptomatic urethritis cases and investigating symptomatic patients^2^. Good test performance depends on strict compliance with all the manufacturers' steps, including collection, transportation, and storage of samples^24^. 

Screening of asymptomatic urethritis cases must be performed using molecular biology techniques, including methods with high sensitivity and specificity, such as polymerase chain reaction and transcription-mediated amplification. These tests, which are based on nucleic acid amplification test (NAAT), allow the usage of male urethral discharge samples or urine, or both, and the identification of one or more pathogens simultaneously in a single sample, depending on the manufacturer. Results are issued discriminating the detected pathogens^24^ ^-^ ^27^. 

For symptomatic urethritis, several tests can be helpful to determine the etiological agents^2^. Bacterioscopy, culture, and molecular biology methods are incorporated into the Brazilian National Health System (SUS) and are available in a varying manner at different levels of health-care service.

In gonococcal urethritis, intracellular Gram-negative diplococci in polymorphonuclear leukocytes can be visualized by bacterioscopy. This method has high sensitivity and specificity in male urethral discharge samples^2^ ^,^ ^24^ ^,^ ^27^. Sample culture in selective medium (e.g., Thayer-Martin or similar) is a technique with good sensitivity and high specificity for the diagnosis of *N. gonorrhoeae* in optimized circumstances with methodological rigor^27^. Briefly, biological samples are cultivated in selective culture medium for *Neisseria sp.* As the culture medium also allows the growth of other Neisseria species, it is essential to perform the Gram stain of typical colonies (visualization of Gram-negative diplococci) and oxidase and catalase tests, which should present positive results when it comes to *N. gonorrhoeae*. For a definitive diagnosis, the colonies may be submitted to biochemical tests (manual or automated) as the particular behavior of bacteria in the presence of several substrates allows the identification of *Neisseria* species^2^ ^,^ ^28^ ^,^ ^29^. The culture also allows the investigation of the gonococcus susceptibility profile to antimicrobials employing techniques for determining the minimum inhibitory concentration (MIC) and has great utility for surveillance purposes and establishing treatment guidelines^24^ ^,^ ^29^. Besides bacterioscopy and culture, molecular biology techniques, such as NAAT, are available in SUS, which present excellent performance for the etiological diagnosis of symptomatic urethritis and are strongly recommended in the etiological treatment of urethral discharge. 

In the absence of the methods mentioned above, it is possible to use tests that suggest the presence of infection but do not specify the infectious agent, such as the positive test for leukocyte esterase in first-void urine or the microscopic examination of first-voided urine sediment presenting more than ten polymorphonuclear leukocytes per field (an increase of 1,000 times)^2^.

## TREATMENT

The recommended treatment for urethritis may or may not depend on laboratory support^2^ (Figure 2). In the presence of urethral discharge complaints, after anamnesis and physical examination, with confirmed urethral discharge and without laboratory support (urethritis without etiological agent identification), the treatment of chlamydia and gonorrhea with azithromycin 1g orally, single-dose, and ceftriaxone 500mg, intramuscular (IM), single-dose, is indicated. In the possibility of performing bacterioscopy (Gram), with visualization of Gram-negative intracellular diplococci, gonorrhea and chlamydia must be treated as described above (ceftriaxone 500mg, IM, single-dose, and azithromycin 1g, orally, single-dose). The presence of Gram-negative intracellular diplococci indicates a gonococcal infection, but does not exclude the possibility of chlamydia infection. In the absence of these findings, only chlamydia must be treated, with azithromycin 1g orally, single-dose. When biological material collection for culture or molecular biology is possible, this must occur at first consultation. The treatment must be instituted immediately, and test results may be analyzed at return visit^2^. 

It is noteworthy that, based on results of the SenGono Project 2015-2017 (described in the subtopic Antimicrobial resistance), the Brazilian treatment guideline recommends nationally dual therapy of ceftriaxone 500mg, IM, single-dose, associated with azithromycin 1g, orally, single-dose, for uncomplicated anogenital gonococcal infection (urethra, cervix, and rectum)^2^.

**FIGURE 3:**
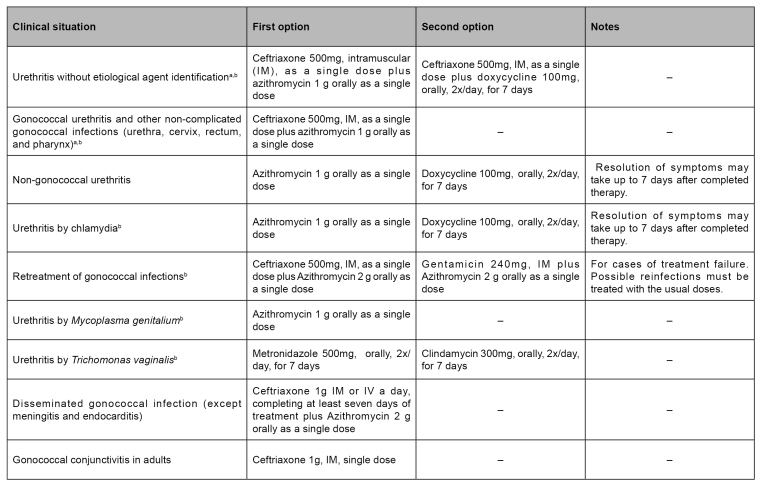
Treatment for urethritis.

**Source:** adapted from the Clinical Protocol and Therapeutic Guidelines for Comprehensive Care of People with Sexually Transmitted Infections^2^.

**Notes:** a) If there is severe allergy to cephalosporins, prescribe azithromycin 2g, four pills, orally, single dose; b) Cases and partnerships should refrain from unprotected sexual intercourse until both have completed their treatment (i.e., after completing multi-dose treatment or for seven days after single-dose therapy).

After seven days of treatment, an appointment must be made to reassess signs and symptoms and to deliver results of culture or molecular biology tests, or both, when performed. Remission of symptoms characterizes healing. In the persistence of symptoms, it is crucial to exclude reinfection, inadequate treatment for chlamydia and gonorrhea, antimicrobial resistance (mainly related to gonococcus and *M. genitalium*), trauma, chemical irritation, or insertion of foreign bodies. In addition, *T. vaginalis* must be treated with metronidazole 500mg, two pills, orally, twice a day, for seven days, and the exams' results must be analyzed to assess the need to institute new treatment in accordance with findings. An appointment for reevaluation must be scheduled after seven days, followed by discharge in the absence of signs and symptoms and referral to specialized services if they persist. Treatment options for urethritis are presented in Figure 3 ^2^. 

## SURVEILLANCE, PREVENTION, AND CONTROL

STIs are among the most common public health problems in Brazil and the world. Primary health care is the first point for STI care, with preventive actions, diagnosis, timely and adequate treatment, and referral of cases that do not fit this care level. To allow integrality of care, services must be organized to promote access to other care levels when necessary^30^.

Anamnesis, identification of different vulnerabilities, and physical examination are essential elements in addressing people with STI. To guarantee the quality of assistance, adherence to treatment, and retention at health care service, the health professional needs to establish a relationship of trust with the person with STI, ensuring privacy and information confidentiality. Professionals must be available for dialogue and provide information on health education, addressing issues such as etiological agents of STI, possible forms of transmission, prevention, diagnosis, and the importance of treatment adherence. In addition, definition of a follow-up strategy, attention to sexual partnerships and access to prevention inputs must be ensured^31^.

Prevention and control of STIs that cause urethritis includes the correct usage of condoms during sexual intercourse; regular screening and testing of asymptomatic people in specific populations; investigation and management of symptomatic cases; institution of immediate treatment, when indicated; and treatment of sexual partners. The therapy, besides having a curative function, also aims at interrupting the chain of infection and preventing complications resulting from urethritis^2^ ^,^ ^31^.

The screening of gonococcus and chlamydia in asymptomatic people is indicated in the following situations: pregnant women under 30 years old, at the first prenatal visit; people living with HIV, at the time of HIV infection diagnosis; people with indication of post-exposure prophylaxis to HIV; people with STI at the time of diagnosis; people who have suffered sexual violence, at the first visit and the four to six weeks afterward visit; people with receptive (passive) anal sex practice without condom use; and people in the use of pre-exposure prophylaxis to HIV, every six months^2^.

It is essential that the sexual contacts of infected people be treated, even when asymptomatic, to interrupt the transmission chain of STIs^3^. Therefore, this information must be passed on to the person with STI while providing communication and support tools until the end of treatment. Confidentiality, absence of coercion, and protection against discrimination must be guaranteed^2^.

In men with gonococcus, *C. trachomatis*, or *M. genitalium* infection symptoms, all previous eight weeks sexual partners need to be examined and treated. In asymptomatic cases, this applies to all sexual partners for the past six months^3^. 

Urethritis are not a national compulsory notification condition; however, urethral discharge syndrome is a mandatory notification condition in some Brazilian states. The Brazilian Ministry of Health has also published an ordinance^32^ that instituted sentinel surveillance sites for male urethral discharge syndrome, which should provide information for the production of official epidemiological data. Furthermore, the goal is to strengthen actions for prevention of STI that causes urethral discharge, subsidize national recommendations for the treatment of urethral discharge syndrome, monitor the gonococcus susceptibility to antimicrobials with the SenGono Project, and research etiological agents of urethral discharge and the antimicrobial resistance of *M. genitalium* ^33^. 

### Antimicrobial resistance

*N. gonorrhoeae* has developed progressive resistance to antibiotics throughout the history of antibiotic therapy, from sulfonamides to fluoroquinolones. After the spread of ciprofloxacin resistance, third-generation cephalosporins have been the basis of treatment in association with azithromycin. The emergence of cephalosporin-resistant gonorrhea will negatively impact the ability of professionals to treat gonorrhea effectively. Gonococcus strains considered multi-drug resistant and extensively resistant to drugs are already found in the Americas, Asia, several European countries, and Oceania^34^ ^-^ ^37^. Therefore, it is essential to continuously monitor antibiotic resistance and encourage research and development of new treatment regimens^38^ ^-^ ^39^.

**FIGURE 4:**
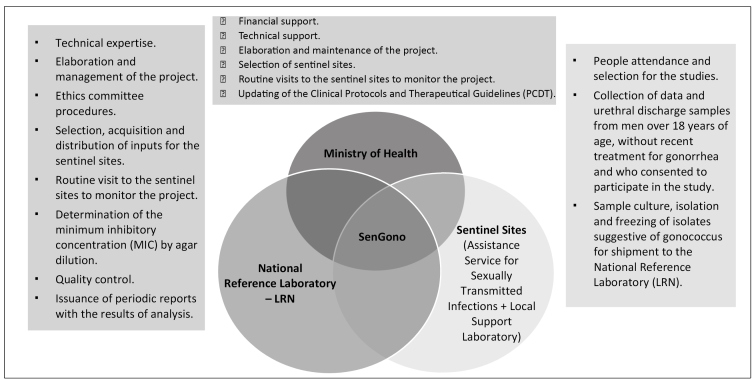
Functions of the Ministry of Health, the National Reference Laboratory, and the Sentinel Sites under the SenGono Project.

**Source:** adapted from the Epidemiological Bulletin no. 27/2020, by the Ministry of Health^32^.

In its "Report on Global Sexually Transmitted Infection Surveillance 2018”, WHO prioritizes the control of gonococcal infections due to the imminent possibility of them becoming untreatable^40^ ^-^ ^41^. Brazil is one of the member countries of global gonococcus susceptibility surveillance program (Gonococcal Antimicrobial Surveillance Programme, GASP)^42^. The activities of GASP in Brazil take place within the SenGono Project, that consists a cooperation between the Ministry of Health, the National Reference Laboratory (Molecular Biology, Microbiology and Serology Laboratory of the Federal University of Santa Catarina), and sentinel sites distributed throughout the country (Figure 4)^33^. 

In the first edition, from 2015 to 2017, high resistance to ciprofloxacin, close to and even higher than 50%, and high susceptibility to third-generation cephalosporins (cefixime and ceftriaxone) were observed in all country's regions^26^. 

The surveillance performed in the SenGono Project has integrated the "National Plan to Combat Antimicrobial Resistance 2019-2023", and is in its second edition (2018-2020), with the expansion to new sentinel sites and evaluation of gonococcus susceptibility to two new antimicrobials (spectinomycin and gentamicin). Also, determination of male urethral discharge etiology and investigation of antimicrobial resistance of *M. genitalium* have been incorporated into this edition^33^.

*M. genitalium* was first identified in 1980 and recognized as an important cause of non-gonococcal urethritis^25^ ^,^ ^43^ ^-^ ^45^. Because the bacteria has no cell wall, antibiotics such as beta-lactams (including penicillins and cephalosporins) are not effective^29^. The introduction of azithromycin, used as single-dose therapy for chlamydia infections, has resulted in clearance of *M. genitalium* from the urogenital tract, eliminating the pathogen without the disease development^25^ ^,^ ^43^ ^,^ ^46^ ^-^ ^50^. However, recent studies indicate an upward trend in prevalence of macrolide-resistant *M. genitalium* infections (transmitted resistance) and cases of induced resistance after azithromycin therapy^3^ ^,^ ^43^ ^,^ ^50^. There is no evidence that azithromycin extended regimen (1.5g) is superior to a single-dose 1g regimen^51^. Moxifloxacin remains highly active against most macrolide-resistant *M. genitalium* strains. However, first clinical cases of moxifloxacin treatment failure have been published^3^ ^,^ ^28^ ^,^ ^43^ ^,^ ^44^ ^,^ ^50^ ^-^ ^53^. Therefore, *M. genitalium* is an emerging problem, requiring frequent surveillance and studies with new diagnostic and treatment options^27^. 

The increase in resistance during recent decades reinforces the importance of etiological diagnosis and adoption of appropriate treatments. In 2016, the World Health Assembly adopted the "Global health sector strategy on Sexually Transmitted Infections, 2016-2021"^38^. This strategy includes the rapid expansion of evidence-based interventions and services to eliminate STI as a public health concern by 2030.
